# Celiac trunk aortic dissection induced by bevacizumab therapy for rectal cancer: A case report

**DOI:** 10.1097/MD.0000000000038882

**Published:** 2024-07-12

**Authors:** Mingming Su, Lili Zhao, Jing Zhou, Xuan Li, Ning Ding

**Affiliations:** a Department of Medical of Oncology, Affiliated Huishan Hospital of Xinglin College, Nantong University, Wuxi Huishan District People’s Hospital, Wuxi, Jiangsu, China.

**Keywords:** bevacizumab, celiac trunk aortic dissection, hypertension, rectal cancer

## Abstract

**Rationale:**

Bevacizumab (Bev) is a humanized monoclonal antibody that targets vascular endothelial growth factor A and is primarily used for the treatment of various solid tumors. Aortic dissection (AD) is a severe vascular disease caused by the tearing of the intimal layer of the aorta or bleeding within the aortic wall, resulting in the separation of different layers of the aortic wall. However, the pathogenesis is not fully understood. Some studies have suggested that Bev treatment is associated with the occurrence of AD.

**Patient concerns:**

A 67-year-old Chinese male was diagnosed with rectal cancer accompanied by liver and lung metastasis. Three days after starting combined chemotherapy with Bev, the patient developed persistent abdominal pain. Abdominal CT scan revealed celiac trunk AD in the abdominal aorta.

**Diagnoses:**

The patient was diagnosed with rectal cancer accompanied by liver and lung metastases. Abdominal CT tomography revealed a celiac trunk AD.

**Interventions:**

Somatostatin combined with valsartan was used to control blood pressure. The patient was subsequently referred for vascular surgery and underwent an abdominal aortic angiography. Conservative treatment was continued.

**Outcomes:**

Three months after the initiation of treatment, follow-up abdominal CT scans showed stability in the condition of celiac trunk AD, with no abdominal pain or hypertension. There were no signs of worsening dissection, aneurysm formation, or inadequate perfusion of end organs.

**Lessons:**

There may be a connection between Bev and elevated blood pressure as well as celiac trunk AD.

## 1. Introduction

Bevacizumab (Bev), a humanized monoclonal antibody targeting vascular endothelial growth factor A (VEGF-A), is primarily used for the treatment of various solid tumors, such as colorectal cancer, non-squamous non-small cell lung cancer, renal cell carcinoma, and breast cancer. This medication functions by inhibiting VEGF-A activity, thereby obstructing the formation of tumor blood vessels and effectively suppressing tumor growth.^[1]^ Despite the pronounced therapeutic efficacy of Bev in clinical treatment, its side effects cannot be ignored. Research indicates that Bev may induce adverse reactions in multiple physiological systems, including the cardiovascular, gastrointestinal, and hematologic systems, potentially affecting the patients’ quality of life. Notably, the side effects of Bev may be influenced by the combination therapy administered to patients.^[2]^

Of particular significance is the occurrence of hypertension, which is one of the most common side effects associated with Bev. Hypertension may be linked to compromised vascular endothelial function, systemic vasoconstriction, and increased peripheral resistance. Additionally, inhibition of VEGF signaling may alter the structure and function of the kidneys, leading to inappropriate sodium excretion and volume overload. Therefore, close attention should be paid to these potential side effects when utilizing Bev for treatment.^[3,4]^

Aortic dissection (AD) is a severe vascular disease caused by the tearing of the intimal layer of the aorta or bleeding within the aortic wall, resulting in the separation of different layers of the aortic wall. Untreated patients have a mortality rate of 1% to 2% per hour after symptom onset.^[5]^ Spontaneous abdominal AD is a rare condition of visceral artery dissection that generally occurs independently of the AD. This condition often presents with acute abdominal or back pain and typically affects middle-aged males, with an average age of 53.1 years. Potential complications include local ischemia and the formation and rupture of arterial aneurysms, typically diagnosed using CT scanning.^[6,7]^ Among the affected visceral arteries, involvement of the superior mesenteric artery is most common. Potential triggers for this condition may include preexisting vascular diseases, hypertension, or pregnancy.^[8]^ The exact etiology of AD remains unclear, but the current understanding implicates factors such as hypertension, genetic predisposition, inflammatory factors, atherosclerosis, and connective tissue disorders.^[9]^ Research indicates that the use of Bev may increase the risk of adverse arterial events, especially cardiac and cerebral ischemia and arterial hypertension.^[10]^ A case report documented a patient undergoing Bev treatment and hepatic artery dissection, leading to the formation of a pseudoaneurysm.^[11]^ This finding suggests a potential association between Bev treatment and the occurrence of arterial dissection. A search using the keywords “Bevacizumab” and “Celiac trunk aortic dissection” in the PubMed database did not yield any relevant cases. To the best of our knowledge, this is the first case of celiac trunk AD caused by Bev. As the global application of Bev continues to increase, in-depth research into its potential side effects has become increasingly crucial.

## 2. Case presentation

A 67-year-old Chinese male was admitted to the hospital on February 19, 2022, because of persistent abdominal discomfort for 1 week. After comprehensive examination, the patient was diagnosed with rectal cancer with liver and lung metastases. The patient had no history of smoking or drinking, and no other chronic medical conditions, except hypertension. On February 16, 2022, colonoscopy conducted at our hospital revealed an irregular intraluminal neoplasm located 15 cm from the anal verge. The texture was fragile and prone to bleeding, and the lumen was narrowed to an extent that further endoscopic examination was not feasible. Pathological examination confirmed it to be rectal mucosal adenocarcinoma. A chest, abdominal, and pelvic CT scan performed on February 21, 2022, showed a rectal tumor with multiple pulmonary metastatic nodules, mild inflammation in the left lower lobe of the lung, and suspected metastatic lesions in the hepatic caudate lobe. Occupying lesions suspected to be associated with rectal cancer were found in the rectal and sigmoid colon regions. Additionally, multiple small lymph nodes were observed in the pelvic and bilateral inguinal regions, with no evidence of AD or aneurysm on the CT scan. Pelvic MRI on February 23, 2022, revealed an occupying lesion in the rectosigmoid region, suspected to be a malignant tumor, with multiple small lymph nodes also observed in the pelvic and bilateral inguinal regions. In summary, the patient was diagnosed with rectal cancer with liver and lung metastases, which made curative surgical treatment difficult. Therefore, the patient was referred to the oncology department on February 24, 2022, and started a systemic chemotherapy regimen on February 26, 2022, consisting of oxaliplatin (Oxa) 200 mg on day 1 plus capecitabine (Cap) (1.5 g) on days 1 to 14. Genetic testing results revealed KRAS G13D mutation at 26.3% and PIK3CA G1007R and E110de1 gene mutations at 13% and 14.4%, respectively, with the tumor being microsatellite stable. To enhance treatment efficacy, the treatment plan was adjusted on March 22, 2022, initiating a new regimen comprising Bev 400 mg on day 0 plus Oxa 200 mg on day 1 and Cap 1.5 g daily on days 1 to 14.

**Figure 1.**
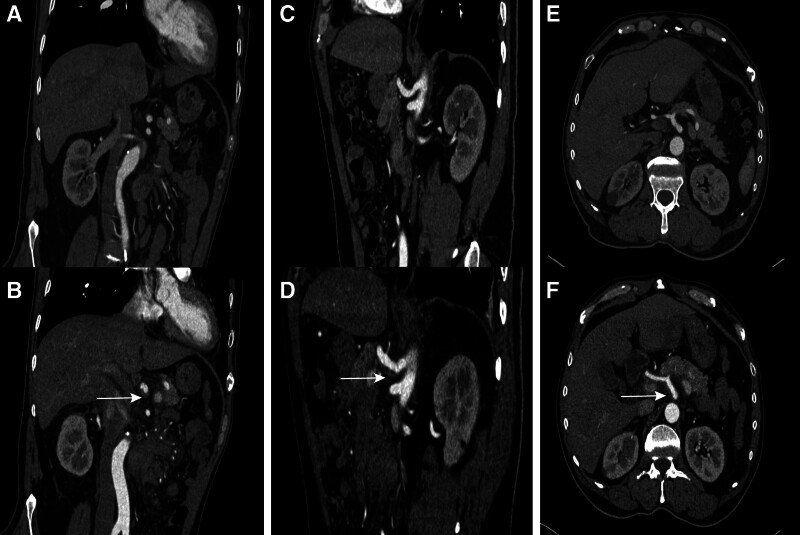
Contrast-enhanced CT scan. From left to right are the comparative images in the coronal, sagittal, and axial planes. Images (A, C, and E) represent the normal images before medication, while images (B, D, and F) represent the abnormal images after medication. Thin white arrows indicate the location of the celiac trunk AD. AD = aortic dissection.

**Figure 2.**
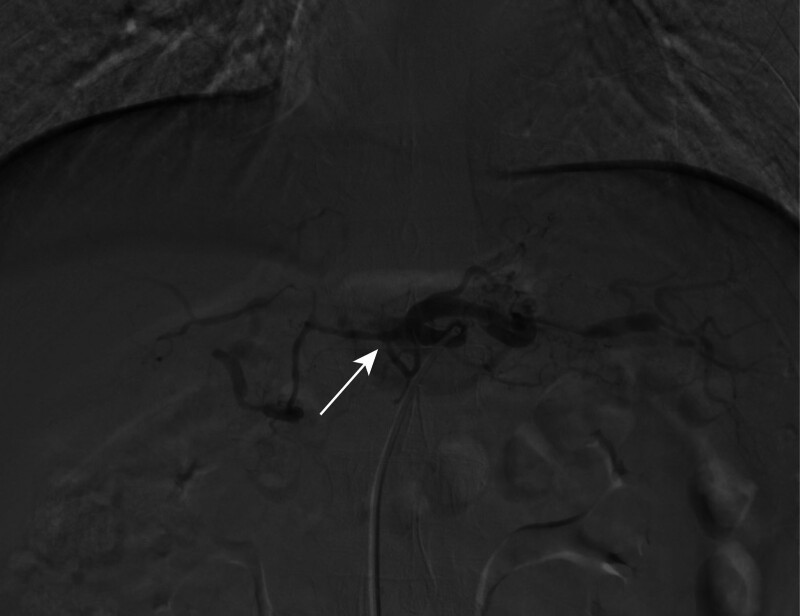
Digital subtraction angiography shows celiac trunk AD. AD = aortic dissection.

In this case report, we describe a patient receiving Bev treatment who developed persistent central abdominal area pain and elevated blood pressure 3 days after treatment without accompanying chest pain, dyspnea, or vomiting. Vital signs on March 27, 2023, showed a normal temperature with a blood pressure of 154/89 mm Hg. Notably, the patient’s blood pressure was 106/65 mm Hg the day before treatment. To manage the blood pressure, the treatment regimen was temporarily supplemented with sublingual nifedipine in conjunction with valsartan. Since the chest and abdominal contrast-enhanced CT scans conducted 1 month ago (Fig. 1A, C, and E) did not reveal any evidence directly linked to abdominal pain, neuropathic pain was initially considered as the potential cause. However, subsequent contrast-enhanced CT and mesenteric angiography ruled out this possibility. Abdominal CT on March 29, 2022 (Fig. 1B, D, and F) revealed findings consistent with celiac trunk AD, intramural hematoma of the superior mesenteric artery, and suspected ruptured bleeding. Additionally, thrombosis in the portal vein and superior mesenteric vein, along with effusion and hematoma in the mesenteric space of the mid-abdomen, were noted. Furthermore, examination of the bilateral femoral, dorsalis pedis, and posterior tibial veins yielded normal results. For further diagnosis and treatment, the patient was transferred to vascular surgery on the same day and received somatostatin therapy along with valsartan for blood pressure management. On April 6, 2022, under local anesthesia, the patient underwent abdominal aortic angiography (Fig. 2), which revealed celiac trunk AD accompanied by local vascular dilation and tortuosity. Owing to the narrowness of the peripheral blood vessels, achieving vascular recanalization and blood flow perfusion was deemed unfeasible. Therefore, the decision was made to abort surgery and transition to conservative treatment. After conservative treatment, the patient’s abdominal pain symptoms gradually subsided. To continue tumor control, it was advised to discontinue Bev, and on April 28, 2022, the patient resumed chemotherapy with Oxa 200 mg and Cap 1.5 g on days 1 to 14, with the process proceeding smoothly.

**Figure 3.**
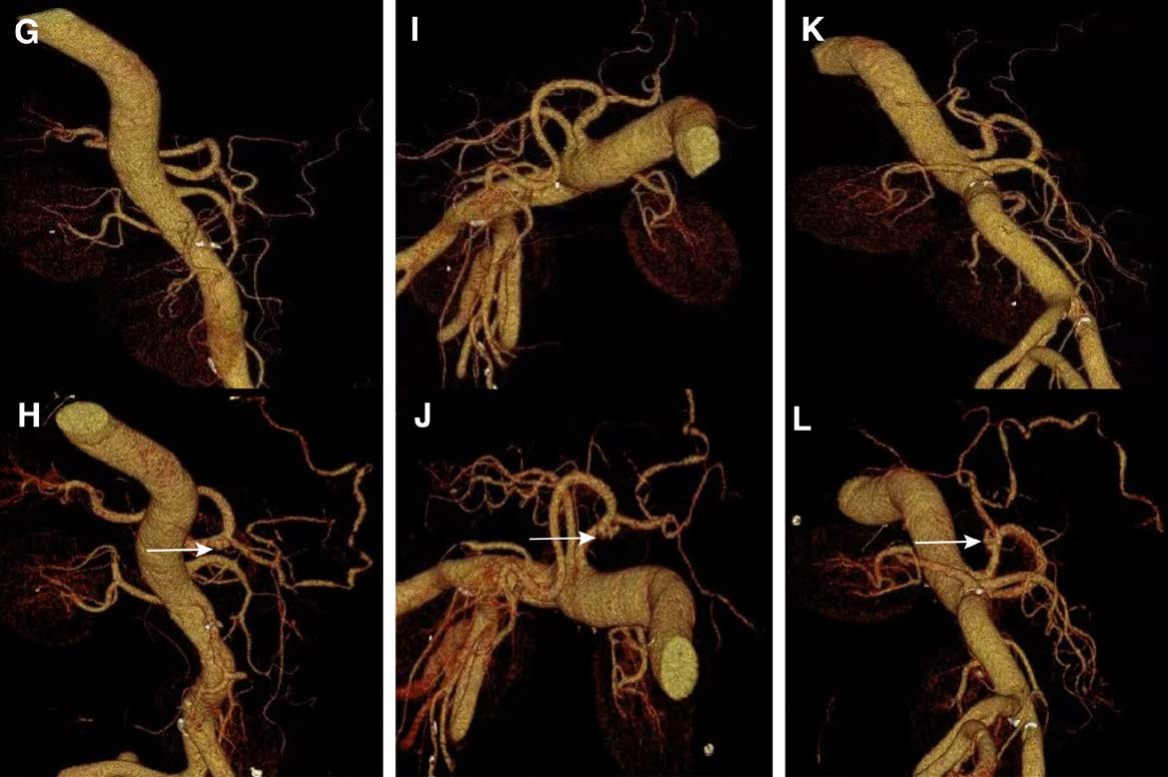
3D reconstructed images from multiple angles showed the celiac trunk AD. Images (G, I, and K) represent the normal 3D reconstructed images, while images (H, J, and L) represent the abnormal 3D reconstructed images. AD = aortic dissection.

**Figure 4.**
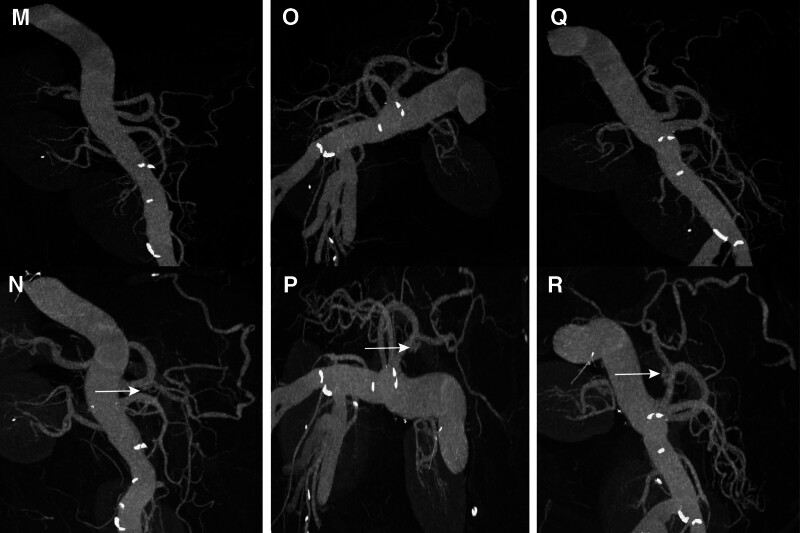
MIP images from multiple angles showed that celiac trunk AD. Images (M, O, and Q) represent the normal MIP images, while images (N, P, and R) represent the abnormal MIP images. AD = aortic dissection. MIP = maximum intensity projection.

An emergency abdominal CT scan revealed celiac trunk AD. Volume Rendering Technology and Maximum Intensity Projection (MIP) demonstrated the formation of celiac trunk AD (Figs. 3 and 4). Linear low-density shadows are observed around the hepatic artery, suggesting fluid accumulation in the surrounding tissues. Additionally, circular filling defects were observed around the portal vein and superior mesenteric vein, with a higher density on plain scans, indicating thrombus formation. The mesenteric space in the mid-lower abdomen showed patchy exudation and a hematoma, possibly indicating signs of internal bleeding. Importantly, the CT scan did not reveal evidence of aortic aneurysm, other thromboses, or impaired perfusion of the terminal organs. The patient did not exhibit any clinical signs of acute limb ischemia.

To manage the patient’s hypertension, the medical team initiated temporary treatment with 10 mg of nifedipine in addition to the existing valsartan regimen. Following detailed discussions with the oncologist, we decided to suspend Bev treatment and continue the chemotherapy regimen based on Oxa and Cap. Three months after the initiation of treatment, follow-up abdominal CT revealed stability in the condition of celiac trunk AD, with no abdominal pain or hypertension observed. There were no signs of worsening dissection, aneurysm formation, or inadequate perfusion of end organs.

## 3. Discussion

AD is an acute vascular condition that typically occurs in elderly individuals. Its hallmark feature involves separation of the inner and outer layers of the aortic wall, creating a false lumen that may exert pressure on the normal aortic channel. The risk of this condition is regulated by various factors including increased vascular wall tension and structural degeneration. Potential triggering factors include hypertension, atherosclerosis, and certain connective tissue disorders.^[12]^ High blood pressure is generally considered the most common predisposing risk factor for AD, and elevated systolic and diastolic pressures are strongly correlated with the risk of AD. Nonlinear dose-response analysis demonstrated a significant increase in the risk of AD with systolic pressure > 132 mm Hg and diastolic pressure > 75 mm Hg. Even within the normal range of blood pressure, the risk of AD showed a dose-dependent increase. These findings further underscore the importance of blood pressure control in preventing AD.^[13]^ Among the anti-angiogenic agents, drugs directly targeting VEGF, such as Bev, show the most significant association with AD. In contrast, drugs such as sorafenib, which indirectly affect VEGF signaling, exhibit a weaker association with arterial dissection.^[14]^ Furthermore, VEGF inhibitors (VEGFI) may increase arterial stiffness, making them more prone to dissection.^[15]^ The Japanese Adverse Drug Event Report Database indicates that the incidence of AD in patients with cancer treated with VEGFI is 0.3%.^[16]^ Among the various classes of VEGFI drugs associated with arterial dissection, Bev is the most closely linked, representing a high proportion of reported cases (up to 35.57%).^[17]^ Bev is a monoclonal antibody targeting VEGF-A that primarily inhibits angiogenesis, tumor proliferation, and metastasis.^[18]^ The known adverse reactions to Bev include hypertension, proteinuria, delayed wound healing, and bleeding. Among these, hypertension is the most common, with an incidence rate of approximately 10% to 30% induced by medication. Yoshimura reported the case of a 68-year-old female with advanced breast cancer who developed AD during Bev treatment. The patient experienced hypertension and proteinuria as side effects of treatment. Severe back pain occurred in the fifth cycle of treatment, and a DeBakey IIIb-type AD was incidentally detected on contrast-enhanced CT in the ninth cycle. This is the first case of AD in a breast cancer patient receiving Bev treatment in Japan.^[19]^ A study reported a case of a female patient who received 8 cycles of Bev and was diagnosed with acute AD after experiencing sudden back pain. Despite undergoing contrast-enhanced CT scans of the chest and abdomen 1 month prior, no abnormalities related to back pain were identified at that time. Subsequent contrast-enhanced CT led to a final diagnosis of acute AD. Unfortunately, the patient died shortly after the exacerbation of chest pain. This case serves as a reminder to clinicians to remain vigilant about the potential risk of acute AD during Bev treatment.^[20]^ Baek documented a case of a 74-year-old male patient from Korea who had a ruptured abdominal aortic aneurysm following intravitreal Bev injections for age-related macular degeneration with neovascularization. Four days after the third intravitreal injection of Bev, a contrast-enhanced CT scan revealed a ruptured abdominal aortic aneurysm with a diameter of 5.8 centimeters, along with bilateral iliac artery aneurysms.^[21]^ Currently, there are 2 hypotheses regarding AD pathogenesis. The early theory suggests that AD begins with rupture of the aortic intima, where high-pressure blood flow through the ruptured intima leads to separation of the aortic media and adventitia. However, a recent hypothesis suggests that AD may originate from an inflammatory response in the aortic media.^[22]^ Although the connection between Bev and abdominal AD remains unclear, studies on the mechanisms of AD formation suggest the involvement of multiple biological processes. These include abnormal phenotypic transition and apoptosis of vascular smooth muscle cells (SMCs), abnormal degradation of the extracellular matrix, endothelial dysfunction, and infiltration of immune cells. The transition of SMCs from a contractile to secretory phenotype leads to thinning and weakening of the aortic wall, increasing the risk of intimal rupture. Additionally, the apoptosis of SMCs affects the contractility and elasticity of the aortic wall. Degradation of the extracellular matrix is another critical factor that weakens the stability and elasticity of the vessel wall, thereby increasing the likelihood of pathology. The infiltration of inflammatory cells, particularly macrophages and other immune cells, may contribute to these pathological changes, resulting in further damage to the vascular wall structure.^[23,24]^ Macrophages are the predominant inflammatory cells in AD tissue. Their infiltration not only signifies a robust immune response to damaged tissue, but may also trigger further aggregation and activation of immune cells, thereby exacerbating the inflammatory state of the aorta. Macrophages induce apoptosis of vascular SMCs, degradation of elastic fibers and the extracellular matrix, endothelial dysfunction, and infiltration of immune cells by secreting pro-inflammatory cytokines and metalloproteinases. When AD occurs, the arterial wall undergoes significant infiltration by macrophages, leading to structural damage.^[23]^ Macrophage infiltration can severely compromise the structure of the aorta, ultimately leading to separation and rupture of the aortic wall.^[15,22]^ The use of VEGFI may increase the risk of arterial dissection, potentially through inhibition of the phosphatidylinositol 3-kinase-AKT signaling pathway, leading to overexpression of matrix metalloproteinase 9 and exacerbation of extracellular matrix degradation. Additionally, VEGFI reduces the production of nitric oxide and prostaglandins, causing hypertension, while increasing the secretion of endothelin-1 to promote vasoconstriction, thereby facilitating the formation of arterial dissections.^[14,25]^ However, the impact of Bev on macrophage infiltration is complex and may depend on the timing of treatment and tumor type. In one study, the early administration of Bev effectively suppressed corneal neovascularization and significantly inhibited macrophage infiltration in the early treatment group. However, in the late treatment group, Bev did not alter macrophage infiltration.^[26]^ In another study, researchers observed that tumor cells treated continuously with Bev developed resistance to Bev at 8 weeks in a mouse model. The number of CD68-positive macrophages increased in these tumors, particularly the number of CD86-positive cells, whereas the number of CD206-positive cells did not change significantly. This indicates that in the tumor microenvironment treated with Bev, the number of certain types of macrophages may increase.^[27]^ Overall, the effect of Bev on macrophage infiltration depends on several factors, including the timing of treatment, tumor type, and tumor microenvironment. In some cases, Bev may inhibit macrophage infiltration, whereas in others, it may increase certain types of macrophages.

In this case, the patient had a history of hypertension but no other known risk factors or family history of AD. Currently, there is no opportunity to obtain pathological specimens of affected arteries to further understand the underlying cause of arterial dissection. Literature suggests that a history of hypertension is not a definitive factor for VEGFI-induced AD; rather, it is the elevation in blood pressure after medication, which is the key factor.^[28]^ Prior to using Bev, the patient had a well-controlled blood pressure. However, after starting Bev treatment, the patient experienced elevated blood pressure and symptoms of celiac trunk AD. This scenario suggests a potential connection among Bev, hypertension, and celiac trunk AD. There are 2 possible explanations. First, Bev may directly contribute to the occurrence of celiac trunk AD. Second, Bev may initially lead to hypertension, which subsequently triggers celiac trunk AD. Considering the mechanism of action of Bev and the complexity of its vascular biology, both hypotheses are plausible. As a VEGFI, Bev may affect the integrity of the blood vessel wall by influencing the vascular stability and repair mechanisms. Additionally, it may increase the risk of AD by modulating blood pressure or by affecting the contractile and relaxant properties of blood vessels. Further clinical studies and case analyses are necessary to confirm these associations.

## Acknowledgments

We are grateful to the patient and all researchers, including the physicians, nurses, and technicians, who participated in this study.

## Author contributions

**Writing – original draft:** MingMing Su.

**Writing – review & editing:** MingMing Su.

**Resources:** LiLi Zhao.

**Supervision:** LiLi Zhao.

**Data curation:** Jing Zhou.

**Formal analysis:** Jing Zhou.

**Validation:** Xuan Li.

**Project administration:** Ning Ding.
